# Relative Validity and Reproducibility of Dietary Measurements Assessed by a Semiquantitative Food Frequency Questionnaire among Chinese Healthy Adults

**DOI:** 10.3390/nu15030545

**Published:** 2023-01-20

**Authors:** Qiumin Huang, Xiaofeng Zhou, Chuqi Zhang, Liyan Huang, Qi Wang, Qinsheng Chen, Huiru Tang, Jingchun Luo, Zhengyuan Wang, Changzheng Yuan, Yan Zheng

**Affiliations:** 1 Human Phenome Institute, School of Life Sciences, Fudan University, Shanghai 200433, China; 2 School of Public Health, Zhejiang University School of Medicine, Hangzhou 310058, China; 3 Department of Nutrition Hygiene, Shanghai Municipal Center for Disease Control and Prevention, Shanghai 200336, China

**Keywords:** food frequency questionnaire, diet record, biomarkers, validity, reproducibility

## Abstract

This study aimed to evaluate the relative validity and reproducibility of a semiquantitative food frequency questionnaire (SFFQ) in adult populations in China. Among the 49 recruited healthy participants (age range: 20–60 years), the relative validity of a 79-item SFFQ was assessed in two ways: (1) by comparing its dietary intake estimates with those from the average measurements of three inconsecutive 24 h dietary records (24-HDRs); and (2) by comparing its estimates of dietary fatty acids with the measured plasma levels of fatty acids. The reproducibility of the SFFQ was evaluated by a comparison of two SFFQ measurements from the same participants collected one year apart. In the relative validity study, the average Spearman correlation coefficient (*r*) was 0.27 among 18 prespecified food group intakes estimated from the SFFQ and the 24-HDRs; nevertheless, that of five food group intakes (e.g., red meat and seafood) was higher (all *r_s_* > 0.40, *p* < 0.05). In addition, a moderate correlation between the SFFQ estimate of polyunsaturated fatty acid intakes (energy-adjusted percentage of total fatty acids) and its plasma level was observed (*r* = 0.42, *p* < 0.05). Regarding the one-year reproducibility of the SFFQ-assessed intakes, the average rank intraclass correlation coefficient (ICC) was 0.35 for the 18 food group estimates. In particular, moderately reproducible estimates of seven food group intakes (e.g., refined grains and red meat, all ICCs ≥ 0.40, *p* < 0.05) were observed. In conclusion, the SFFQ provides valid and reproducible estimates of dietary intakes for various food groups in general and performs well as a potential tool for estimating habitual dietary intakes of some unsaturated fatty acids.

## 1. Introduction

Nutritional epidemiologic studies have identified the associations of dietary factors with various health outcomes related to cardiometabolic disease, immune disorders, and neurological diseases [1,2,3]. The quality of such evidence is largely affected by the validity and reproducibility of habitual diet estimates. Food frequency questionnaires (FFQs), which have been developed as the primary method of dietary assessment in large-scale studies [4], measure individual intakes of both foods and nutrients over an extended period of time and are cost-efficient compared with diet records [5,6]. Most FFQs center on frequencies of food intakes during a defined period such as the past year, and some further focus on the specific portion size of food items designed according to available and appropriate food lists and eating habits of the specific population concerned. However, measurement errors ineluctably occur in these questionnaires, because the assessment methods rely on participants’ ability to recall [4]. Meanwhile, 24 h dietary recalls (24-HDRs), in which participants need to report food and beverage consumption in real time for several consecutive or nonconsecutive days, have been widely considered as another method to evaluate the validity of FFQs [7,8,9].

As the self-reported methods may give rise to bias, and dietary biomarkers provide reliable and objective measurement, emerging studies have utilized biomarkers of diets to explore the dietary effects on health [10]. Various biomarkers have been used to partially validate the estimated dietary intakes from self-reports. For example, the doubly labeled water technique is the standard method for evaluating energy intake, and 24 h urine nitrogen levels are used for the assessment of protein intake [10]. Moreover, circulating fatty acids, especially those not produced in the human body, such as eicosapentaenoic acid (EPA) and docosahexaenoic acid (DHA), are common biomarkers of dietary fat and fatty acid consumption [11]. Thus, these diet-derived biomarkers have been widely used as objective references in FFQ validation studies [12,13,14].

Over the past decades, previous studies have applied FFQs to estimate the dietary intakes of specific age groups [15,16,17] or in different geographic areas [18,19,20] in Chinese populations. These studies have consistently demonstrated that inappropriate FFQs might lead to measurement errors that significantly impede estimating dietary associations [21]. In addition, as changes may occur easily in dietary habits due to the physiological, social, and cultural backgrounds of participants and the development of the food market [22], the validity and reliability assessment of an FFQ should be assessed every a few years [4].

In this study, the relative validity and reproducibility of a semiquantitative FFQ (SFFQ) were assessed in healthy Chinese adults. The relative validity of the SFFQ was evaluated by comparing its estimates with those from 24-HDRs and available plasma fatty acid biomarkers, and the one-year reproducibility was examined by a comparison of repeated measurements.

## 2. Materials and Methods

### 2.1. Study Participants

The International Human Phenome Project (phase I) (IHPP-I) and the Central China Cohort (CCC) are population-based studies conducted in Chinese participants in the Shanghai and Xinmi areas, respectively. Participants in both studies were comprehensively interviewed on their lifestyle factors and medical history, and their fasting plasma samples were collected at enrollment. We invited participants aged 20–60 years in the IHPP-I and CCC who did not plan to change their diet or physical activity substantially to participate in our study. We excluded those with diseases that affect nutritional status and dietary intake (e.g., diabetes, hypertension, dyslipidemia, cancer, and gastrointestinal diseases) or with major neurological disorders affecting cognition.

The eligible participants were asked to record their 24 h dietary consumption for three days and complete the SFFQ four weeks apart to avoid artificially high correlations. Data were collected from 49 eligible participants (IHPP-I: *n* = 27; CCC: *n* = 22), and they had a reasonable value for total daily energy intake (600–3500 kcal/day). In addition, the participants from the IHPP-I completed the same SFFQ twice (SFFQ1: the first SFFQ collected during the enrollment period of the project; SFFQ2: the second SFFQ collected in this study; approximately one year apart) (Figure S1).

### 2.2. Ethics Approval

The ethics approval for this study protocol was obtained from the internal review board of Fudan University with number FE20064 on 16 October 2020. All the participants provided their written informed consent before any study-specific investigation.

### 2.3. Semiquantitative Food Frequency Questionnaire

The SFFQ was developed based on previous questionnaires of usual dietary intake for middle-aged and older adults in Shanghai that were validated about 15 years ago [20,23]. Due to a younger-targeted population in the current study and the changed dietary habits among Chinese people during the past decades [24], a modified SFFQ was developed, supplementing more details in consumption frequencies and the portion size specified for each food item (see Supplementary Materials). This SFFQ contained 79 items, including 61 foods (e.g., rice, pork, and fish), 11 condiments (e.g., oil, salt, and sugar), and 7 dietary supplements (e.g., multivitamins, calcium, and vitamin D). Participants were asked about their average habitual consumption of the specified amount of each item during the past year. The consumption was measured according to 9 predefined frequency categories ranging from “<1 time per month” to “≥6 times per day” for food items and dietary supplements and 6 possible frequency categories ranging from “never/almost never” to “≥1 time per day” for each type of condiment. Participants were suggested to report up to 5 additional foods consumed more than once per week that were not included in the SFFQ. Frequencies reported on the SFFQ were converted into daily frequencies and multiplied by contents per portion size for the calculation of food intakes. Food intakes were then summed into 18 predefined food groups based on their diverse effects on health, including refined grains, whole grains, tubers, legumes, vegetables, salty vegetables, mushrooms, fruit, red meat, poultry, processed meat, animal organs, seafood, eggs, dairy products, nuts, desserts, and beverages. Based on the Chinese Food Composition Table [25], nutrient intakes were calculated by multiplying food intakes and the nutrient composition for the portion size specified for each item and were then summed across all foods and supplements to obtain the total intake for each individual. The SFFQ-measured dietary percentages of monounsaturated (MUFA) and polyunsaturated fatty acids (PUFA), related to dietary total fatty acids (TFA), were calculated as the MUFA or PUFA value divided by the TFA value, respectively.

### 2.4. 24 H Dietary Records

Three nonconsecutive 24-HDRs were administered on 2 weekdays and 1 weekend day within the same week (Wednesday, Thursday, and Sunday). Each participant was provided with detailed instructions with examples of completed forms, a food scale to take measurements prior to the first day’s dietary record, and a food diary to record consecutive 24 h consumption of foods, beverages, and supplements. Recipes of foods and ingredients for mixed dishes and labels of store-brand products were also collected. Through a face-to-face interview, a trained dietician checked the daily food diary with each participant for completeness and accuracy on the next day of each diet recording. Based on the three-day data collection, the average daily intake of food and nutrients was calculated for comparison with measurements using other methods. Consistent with those in the SFFQ, foods reported in the 24-HDRs were categorized into 18 groups, with their intakes summed within each group. Data on food nutrients were extracted from the Chinese Food Composition Table [25], of which foods unincluded in the database were matched with an adjacent alternative.

### 2.5. Biomarker Measurements

Four circulating biomarkers, i.e., MUFA and PUFA ratios (percentage of TFA) and DHA and EPA concentrations (μmol/L), were available among IHPP-I participants. Their circulating levels were measured using the fasting plasma samples collected about 7 months after the SFFQ1. The ratio of MUFA to TFA and that of PUFA to TFA were determined based on a 600 MHz AVANCE III nuclear magnetic resonance spectrometer equipped with a BBI probe (Bruker Biospin GmbH, Rheinstetten, Germany). DHA and EPA concentrations were quantified using a liquid chromatography–mass spectrometry system with multiple reaction monitoring (Shimadzu Nexera X2, Kyoto, Japan, coupled with SCIEX QTRAP 6500 plus, Framingham, MA, USA). The methods used for biomarker assessment have been described in detail in previous studies [26,27].

### 2.6. Statistical Analysis

The distributions of total daily intakes of food groups, energy, and nutrients were presented as medians and interquartile ranges. The energy-adjusted consumption of foods and nutrients was calculated using the residual method (regressing food and nutrient intakes on total energy intake with a reference energy level of 2000 kcal/day for men and 1800 kcal/day for women) [28]. Because the distributions of most food groups and nutrients were skewed, values were log-transformed after setting “zero” values to fixed non-zero ones (0.0001 unit/day) before the calculation of the residuals [16]. Subsequent analysis of correlations was based on the ranks of the unadjusted values and energy-adjusted values.

Bland–Altman plots were used to visualize the agreement of measurements between the SFFQ and the 24-HDRs. Specifically, the differences between intake estimates from the two dietary assessment methods were plotted against the average value of the differences. In calculating Spearman correlation coefficients (*r*_s_) for the validity estimates of the SFFQ, we utilized the average measurements of three 24-HDRs and the measured plasma levels of fatty acids as comparison methods. Given that EPA and DHA are recommended markers of seafood intake in plasma [29,30,31], the seafood intake via the SFFQ was compared with EPA and DHA concentrations in plasma. For the reproducibility analysis of the SFFQ, rank intraclass correlation coefficients (ICCs) were calculated using a random-effect analysis of variance among participants with two SFFQ measurements. In general, *r*_s_ or ICCs of 0.40 or higher represented a moderate validity or reproducibility [4]. All statistical analyses were performed using R v4.1.1 (https://www.R-project.org, accessed on 20 December 2022).

## 3. Results

### 3.1. Characteristics of Participants

Among the 49 participants (18 men and 31 women) enrolled in the study, the median age was 29.0 years; their median weight and body mass index were 60.9 kg and 21.3 kg/m^2^, respectively. More than half of the participants (67.3%) had a bachelor’s degree or higher, 55.1% were married, and only one current smoker was included. By design, IHPP-I participants were younger than those recruited from CCC, and their other characteristics were comparable (Table S1).

### 3.2. Dietary Intakes

The distributions of daily food group intakes assessed via the SFFQ and the average 24-HDR are shown in Table 1. Compared with the 24-HDRs, the SFFQ had higher median estimates for most food group intakes, except those of refined grains and whole grains. In particular, the median intakes of food groups consumed seasonally (e.g., vegetables, mushrooms, and fruit) assessed with the SFFQ were higher than those obtained from the 24-HDRs. For nutrients, the SFFQ also provided higher median estimates for most macronutrients, vitamins, and minerals compared with the 24-HDRs (Table S2).

For intake assessments defined by participant sources, the median differences between the two methods were slightly smaller among IHPP-I participants than CCC ones (Tables S3 and S4).

### 3.3. Relative Validity

**Figure 1:**
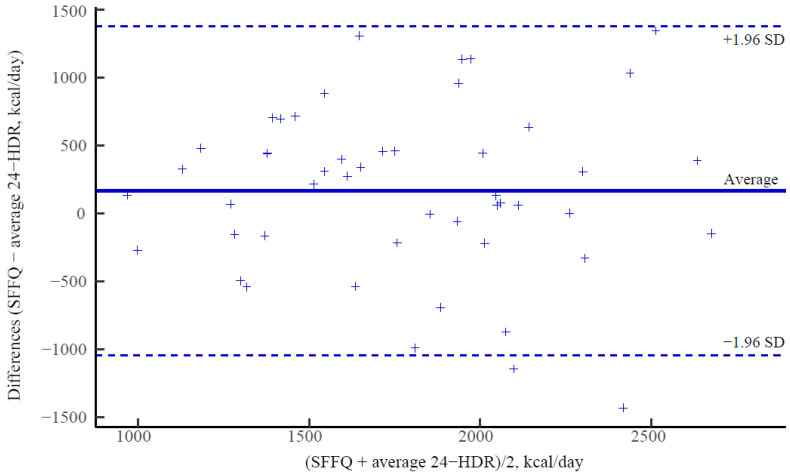
Bland–Altman plot of total daily energy intake reported by each participant. The Y axis is the difference between estimate of total energy intake measured by SFFQ and average three 24-HDRs. The X axis is the average energy intake of the two methods. The central solid horizontal line represents the average difference between the two methods for all participants, and the dashed lines represent the ±1.96 standard deviation (SD) of the average difference.

In the Bland–Altman plot, we observed that the measurements of total energy intake using SFFQ and the average 24-HDR were in a good agreement in general, and the difference in measurements of most participants lay relatively close to the average difference (i.e., the difference in 96% (47/49) participants lay within the 1.96 standard deviation of the average, Figure 1). Similar agreements of the measurements between the two dietary assessments were indicated for almost all assessed food groups and nutrients.

**Figure 2:**
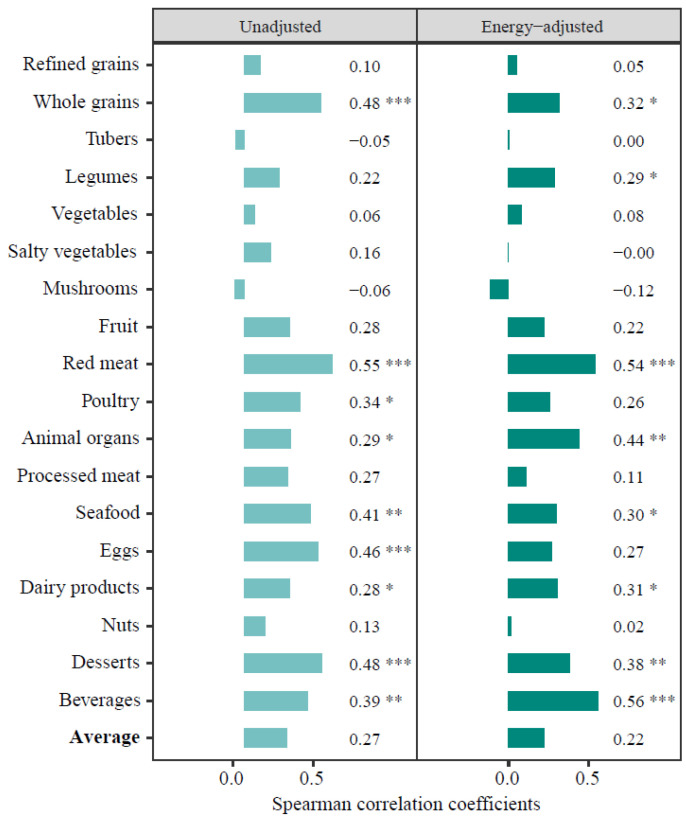
Spearman correlation coefficients (*r*_s_) of food group intakes estimated by the SFFQ and the 24-HDRs. Energy-adjusted: total energy intake was adjusted. * *p* < 0.05; ** *p* < 0.01; *** *p* < 0.001.

Comparing food group intakes assessed by the SFFQ with that by the 24-HDRs, we observed that the average unadjusted *r* was 0.27 and was higher in measurements of whole grains, red meat, seafood, eggs, and desserts (all *p* < 0.05) (Figure 2). For additional food group intakes, correlations between the two methods were fair for poultry and dairy product estimates (*r*_s_ between 0.30 and 0.40), whereas weak correlations were shown for refined grains, tubers, mushrooms, and vegetables (*r*_s_ ≤ 0.10). With the adjustment for total energy intake in both methods, their correlations for most food group estimates slightly decreased, indicated by the average *r* of 0.22. For nutrient intakes, the energy-adjusted correlations between the two methods were moderate for protein, retinol, niacin, and selenium estimates (*r*_s_ ≥ 0.45, all *p* < 0.05) (Figure S2). For intake estimates via the two methods across participant sources, stronger correlations between the two methods were observed among IHPP-I participants in both unadjusted and energy-adjusted estimates, compared with those in CCC participants (Figures S3–S6).

**Figure 3:**
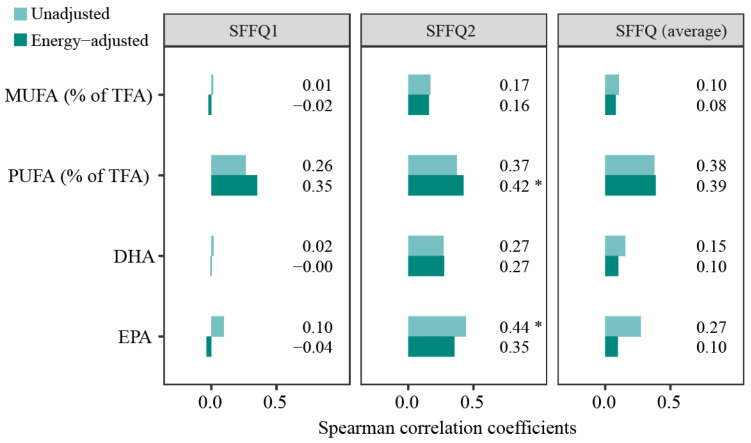
Spearman correlation coefficients (r_s_) of dietary intakes estimated by the SFFQ in different periods against the intake biomarkers. * *p* < 0.05. SFFQ1: data showed the habitual intakes for the last year of the biomarker measurements. SFFQ2: data showed the habitual intakes for the same year of the biomarker measurements. SFFQ (average): data showed the average levels between SFFQ1 and SFFQ2. Energy-adjusted: total energy intake was adjusted. MUFA (% of TFA): the percentage of monounsaturated fatty acids to total fatty acids. PUFA (% of TFA): the percentage of polyunsaturated fatty acids to total fatty acids. DHA: docosahexaenoic acid, EPA: eicosapentaenoic acid.

Utilizing the measured plasma levels of fatty acids as the reference values, we observed close associations between these biomarker levels and the respective intake estimates via the SFFQ2, though not in the SFFQ1 data or the average levels by two SFFQ assessments (Figure 3). For SFFQ2-assessed intakes showing the habitual intakes for the whole year when the biomarkers were measured, moderate correlations were observed between PUFA intake (% total TFA) with its plasma levels, with *r_s_* varying from 0.37 to 0.42 (*p* < 0.05) after accounting for total energy intake. However, the unadjusted and energy-adjusted correlations between MUFA intake (% total TFA) and its plasma levels were poor (both *r*_s_ < 0.20). When the seafood intake was compared with plasma DHA and EPA concentrations, the correlations for EPA decreased from moderate (*r* = 0.44, *p* < 0.05) to fair (*r* = 0.35) after total energy intake was taken into consideration, whereas the correlations for DHA remained constantly fair (both *r_s_* = 0.27).

### 3.4. Reproducibility

**Figure 4:**
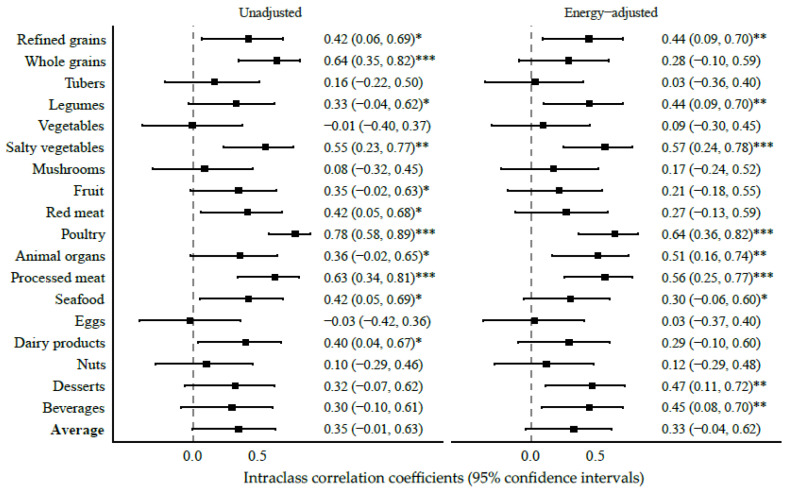
Intraclass correlation coefficients (ICCs) and 95% confidence intervals of food group intakes estimated by the SFFQ in the International Human Phenome Project (phase I). * *p* < 0.05; ** *p* < 0.01; *** *p* < 0.001.

For intake assessments of food groups between SFFQ1 and SFFQ2, the average ICC was 0.35 for unadjusted estimates and slightly decreased by 0.02 for energy-adjusted estimates (Figure 4). Specifically, moderate to high reproducibility was observed between the repeated estimates of refined grains, legumes, salty vegetables, poultry, animal organs, processed meat, and seafood, with the ICCs of unadjusted and energy-adjusted intakes ranging from 0.30 to 0.78 (all *p* < 0.05). In contrast, poor reproducibility was observed between repeated estimates of vegetable and egg intakes (both ICCs < 0.01). For nutrient estimates, the ICCs had an average of 0.31 for the unadjusted intakes, and they were, in general, consistent when energy intake was adjusted in the correlation. Nevertheless, the repeated estimates of seven nutrient intakes (i.e., fat, thiamin, niacin, manganese, TFA, MUFA, and PUFA) were moderately reproducible (ICCs ≥ 0.40, all *p* < 0.05) in both their unadjusted and energy-adjusted estimates (Figure S7).

## 4. Discussion

This study evaluated the performance of a modified SFFQ in capturing the usual dietary intakes of healthy Chinese adults. In general, moderate validity was observed for various food groups and nutrient intakes via the SFFQ compared to the measurements from the 24-HDRs. Meanwhile, the plasma levels of unsaturated fatty acids, i.e., PUFA and EPA, were well correlated with the corresponding SFFQ-assessed intakes for the year when the biomarkers were measured. In addition, reasonable reproducibility was obtained for dietary intakes between repeated assessments via the SFFQ collected one year apart.

Misestimation of food and nutrient intakes on FFQs or 24-HDRs has been consistently found in self-reporting methods [19,32]. Similarly, under- and over-estimating of daily intakes for food groups and nutrients obtained from the SFFQ were observed compared with that from the 24-HDRs, and over-estimating was more common than under-estimating in this study. Specifically, the food intakes usually consumed (e.g., refined food and red meat) and the three energy-yielding macronutrients were slightly different between the two dietary assessment measurements, but greater different estimations of intakes were shown among those episodically consumed foods (e.g., vegetables and fruit) and their relating nutrients (e.g., vitamin A and vitamin C). Misclassifying food intakes in the 24-HDRs into the same or adjacent categories of the SFFQ might be a possible explanation for these different intakes. For instance, bread types can be clearly clarified into white or wholegrain types assessed by the 24-HDRs under the guidance of trained dieticians, but probably failed to match with the respective item of the SFFQ when participants were not familiar with their bread types. In addition, the perception of the specific size predefined in the SFFQ for quantifying each serving varied significantly, causing inaccurate estimations in the SFFQ, but not in the 24-HDRs. Different estimates, thus, can be obtained from two methods, playing roles in affecting the relative validity and reproducibility of the FFQ. Nevertheless, the good agreement between the two assessment measurements was indicated at individual level, as reasonably proportional biases for almost all assessed dietary factors were illustrated by Bland–Altman analyses.

Currently, standards for evaluating FFQs’ validity are not well defined yet. Due to the independent bias caused by FFQs [4], the 24-HDRs, which were obtained from real-time interviews by trained dietitians, have been frequently used as a superior measurement for comparison in many validation studies. In general, the correlation coefficients of dietary estimates between FFQs and 24-HDRs ranged from 0.15 to 0.90, and the FFQ validity particularly presented a better performance in dietary assessments of food groups than those of nutrients [19,20,23,33,34]. In the Shanghai Men’s Health Study (SMHS), the correlation coefficients between the two methods ranged from 0.39 to 0.64 for food groups, 0.39 to 0.53 for macronutrients, and 0.38 to 0.52 for micronutrients [23]. Among Shanghai participants, our results compared well with these estimates for macronutrients in SMHS, but not that well for food groups or micronutrients. This may reflect the changeable dietary compositions of individuals in the same geographic region. Furthermore, the validity of FFQs also depends on the representative food item data and factors in the 24-HDRs, such as the recall period and the collection number of record days [35]. For example, if the 24-HDRs were repeated each season, covering weekdays and weekends, the effect of daily and seasonal variation in dietary intake might be minimized. Then, the average 24-HDR may become a relatively ideal reference method for the validity of FFQs to estimate the usual intakes during the past year. In the current study, we did not collect the 24-HDRs across four seasons, which might explain the relatively low validity of the SFFQ for some food groups consumed seasonally or episodically and their related nutrients.

Circulating biomarkers have been commonly utilized to reflect the long-term intake of dietary composition, especially those not produced within the body. Fatty acid biomarkers, particularly unsaturated fatty acids, frequently serve as objective reference measurements for the validation of other dietary methods to estimate the respective intake [14,31,36,37]. In a study conducted on 125 middle-aged adults in southern China, the correlation coefficients for MUFA, PUFA, EPA, and DHA intakes assessed with the FFQ ranging from −0.08 to 0.37 compared to their corresponding biomarker level, with a poor correlation for the saturated fatty acid [38]. For similar plasma estimates in our study, the diet–biomarker correlations also varied across fatty acids. Similarly, the poorest correlation for MUFA was shown due to the endogenous synthesis [39,40]. Regarding the relatively appropriate incorporation and washout of biomarkers in response to dietary intakes, FFQs demonstrated an advantage over food records in terms of measuring habitual intakes rapidly [11]. To quantify diet–biomarker correlations, an appropriate reference time frame for dietary assessment measurements is crucial, because stronger diet–biomarker correlations were observed when the SFFQ and the measured biomarkers were targeted to assess intake and plasma levels for the same period.

The reproducibility of FFQs estimated by ICCs generally varied from 0.30 to 0.80 for both food groups and nutrients in previous studies conducted among Chinese adults, and the coefficients were wider and lower in nutrients than those in food groups [18,19,33]. The high reproducibility for the egg intake was shown previously, in which the ICC was 0.62~0.67 in Nanjing adults [18] and 0.41~0.44 in Taizhou individuals [19] due to the stable calculation method (counting an egg with a weight of 50~60 g as one serving). However, this method of calculation may impede measuring the number of eggs consumed, causing the relatively low reproducibility for FFQ-assessed egg intakes. Details obtained in 24-HDRs might help to explain the low reproducibility: participants sometimes only ate egg white and evaluated the egg intake from a dish-based meal shared with family members. For nutrients, in our present study, the ranges of ICCs were wider, and the ICC estimates were lower compared with those of 0.65~0.88 for 13 nutrients in Nanjing adults [18] and 0.28~0.62 for 15 nutrients in Taizhou individuals [19]. Besides the macronutrients and vitamins commonly analyzed in validation studies, other types of micronutrients, including minerals and amino acids, which accounted for a significant part of low ICCs (0.10~0.20), might partially result in the low average reproducibility of the SFFQ at nutrient levels in our study. Moreover, various time intervals between repeated SFFQ’s measurements, from 15 days to several years, have been reported in previous studies [15,41]. Although independent data are more likely to be obtained in long intervals, the habitual diet might change between the two measurements. Thus, the one-year interval between our SFFQ’s measurements may also affect its reproducibility for dietary estimates.

There are some limitations in our study. First, the 24-HDRs collected only covered one week, which might fail to capture the intakes of episodically and rarely consumed foods. Second, alcohol was not included in the SFFQ, which might influence the estimate of total energy intake, though no participant consumed alcohol obtained from the 24-HDRs. Third, women tend to have more heterogeneous dietary habits than men [42]; however, we did not analyze the influence of gender on the relative validity and reproducibility of the SFFQ due to the relatively small sample size. Additional evaluation studies with larger sample sizes using more nutritional biomarkers as standards can be utilized to provide more information on the validity of self-reported methods.

## 5. Conclusions

In summary, the 79-item SFFQ provides an acceptably valid and reproducible assessment of various food group and nutrient intakes among healthy Chinese adults. In addition, the SFFQ is suitable for the habitual dietary assessments of some unsaturated fatty acids. Further studies with a larger sample size are suggested to collect multiple 24-HDRs and biomarkers over a study period.

## Figures and Tables

**Table 1:** Median (interquartile ranges) food group intakes estimated by the SFFQ and the 24-HDRs.

Food Groups	SFFQ	24-HDRs
Refined grains, g/day	177.1(120.3, 270.3)	193.3(150.0, 268.3)
Whole grains, g/day	14.3(10.0, 57.1)	26.7(0.0, 110.0)
Tubers, g/day	57.1(14.3, 85.7)	33.3(15.0, 73.3)
Legumes, g/day	19.6(12.6, 36.4)	15.8(4.7, 20.3)
Vegetables, g/day	260.0(173.1, 416.0)	97.7(53.3, 170.0)
Salty vegetables, g/day	1.7(1.7, 5.0)	0.0(0.0, 0.0)
Mushrooms, g/day	14.3(10.0, 35.7)	3.3(0.0, 10.0)
Fruit, g/day	156.9(96.9, 244.8)	66.1(36.7, 182.2)
Red meat, g/day	47.9(14.3, 78.6)	31.7(6.7, 65.7)
Poultry, g/day	28.6(5.0, 42.9)	16.7(0.0, 54.3)
Animal organs, g/day	3.3(3.3, 10.0)	0.0(0.0, 5.0)
Processed meat, g/day	5.0(1.7, 5.0)	0.0(0.0, 5.0)
Seafood, g/day	26.1(14.7, 44.2)	0.0(0.0, 23.3)
Eggs, g/day	62.0(57.4, 66.0)	40.0(16.7, 73.0)
Dairy products, g/day	211.2(87.4, 312.4)	76.7(0.0, 133.3)
Nuts, g/day	3.6(2.5, 3.6)	3.1(0.0, 13.3)
Desserts, g/day	17.5(12.5, 39.4)	8.3(0.0, 21.3)
Beverages, ml/day	83.8(47.7, 121.0)	0.0(0.0, 66.7)

Abbreviations: SFFQ: semiquantitative food frequency questionnaire; 24-HDRs: 24 h dietary recalls.

## Data Availability

Data described in the manuscript, raw data, and analytic code will be made available upon pending request.

## Supplementary Materials

The following supporting information can be downloaded at: https://www.mdpi.com/article/10.3390/nu15030545/s1, Table S1: Characteristics of participants in the validation study; Table S2: Median (interquartile ranges) nutrient intakes estimated by the SFFQ and the 24-HDRs; Table S3: Median (interquartile ranges) food group intakes estimated by the SFFQ and the 24-HDRs by participant sources; Table S4.:Median (interquartile ranges) nutrient intakes estimated by the SFFQ and the 24-HDRs by participant sources; Figure S1: Design of the validation study; Figure S2: Spearman correlation coefficients of nutrient intakes estimated by the SFFQ and the 24-HDRs; Figure S3: Spearman correlation coefficients of food group intakes estimated by the SFFQ and the 24-HDRs in the International Human Phenome Project (phase I); Figure S4: Spearman correlation coefficients of food group intakes estimated by the SFFQ and the 24-HDRs in the Central China Cohort; Figure S5: Spearman correlation coefficients of nutrient intakes estimated by the SFFQ and the 24-HDRs in the International Human Phenome Project (phase I); Figure S6: Spearman correlation coefficients of nutrient intakes estimated by the SFFQ and the 24-HDRs in the Central China Cohort; Figure S7: Intraclass correlation coefficients (ICCs) and 95% confidence intervals of nutrient intakes estimated by the SFFQ in the International Human Phenome Project (phase I). Additional file: A semiquantitative food frequency questionnaire.
