# Supplementary material for: Relative Validity and Reproducibility of Dietary Measurements Assessed by a Semiquantitative Food Frequency Questionnaire among Chinese Healthy Adults
Source: Nutrients. 2023 Jan 20;15(3):545. doi: 10.3390/nu15030545 (PMC9920913; doi:10.3390/nu15030545)
Supplement: Supplementary file 1 [file nutrients-15-00545-s001.zip › nutrients-2144401-supplementary.pdf]

Table S1. Characteristics of participants in the validation study [a].

Variables	Total (n=49)	IHPP-I (n=27)	CCC (n=22)
Age at enrollment, years	29.0(26.0, 45.0)	27.0(24.0, 29.0)	47.0(30.0, 55.8)
Men, %	18(36.7)	13(48.1)	5(22.7)
Bachelor or higher degrees, %	33(67.3)	25(92.6)	8(36.4)
Married, %	27(55.1)	6(22.2)	21(95.5)
Current smoker, %	1(2.0)	0(0.0)	1(4.5)
Weight, kg	60.9(54.3, 69.9)	59.6(53.1, 69.3)	61.7(57.6, 69.4)
Body mass index, kg/m^2	21.3(20.4, 25.6)	22.2(20.1, 25.1)	23.7(22.2, 26.4)

Abbreviations: IHPP-I: International Human Phenome Project (phase I); CCC: Central China Cohort.

[a] Data were expressed as median (interquartile ranges) for continuous variables and no (percentage) for categorical variables.

Table S2. Median (interquartile ranges) nutrient intakes estimated by the SFFQ and the 24-HDRs.

Nutrients	SFFQ	24-HDRs
Total energy, kcal/day	1816.8(1537.3, 2140.8)	1548.4(1359.7, 2021.4)
Carbohydrate, g/day	211.2(170.3, 264.4)	189.2(142.8, 268.5)
Fat, g/day	76.6(57.3, 91.8)	63.7(47.0, 79.7)
Protein, g/day	72.1(56.0, 91.4)	63.8(49.1, 78.9)
Fiber, g/day	14.0(10.2, 19.0)	9.2(6.6, 12.3)
Cholesterol, mg/day	477.2(416.0, 550.1)	225.4(151.1, 350.3)
Vitamin A, μgRE/day	1187.3(749.5, 1384.6)	299.1(186.7, 454.5)
Total carotene, μg/day	2166.8(1719.1, 3312.9)	829.0(344.2, 1946.5)
Retino1, mg/day	698.6(515.1, 1117.6)	89.2(42.4, 182.6)
Thiamin, mg/day	0.9(0.7, 1.1)	0.6(0.4, 0.8)
Riboflavin, mg/day	1.5(1.2, 1.8)	0.8(0.6, 1.0)
Niacin, mg/day	15.6(11.8, 23.0)	13.5(8.6, 15.2)
Vitamin C, mg/day	110.8(85.2, 167.0)	51.7(31.5, 86.4)
Vitamin E, mg/day	31.2(21.6, 39.4)	14.9(10.3, 19.6)
Calcium, mg/day	899.6(745.5, 1108.1)	386.0(329.3, 501.6)
Phosphorus, mg/day	1261.6(1027.7, 1517.7)	834.1(666.1, 1058.2)
Potassium, mg/day	2993.5(2255.5, 3453.9)	1584.0(1286.8, 2022.1)
Sodium, mg/day	7314.1(6856.7, 7695.8)	1431.0(673.4, 1793.8)
Magnesium, mg/day	380.6(296.1, 448.2)	227.7(168.3, 299.6)
Iron, mg/day	28.4(22.6, 32.7)	14.8(11.3, 17.9)
Zinc, mg/day	11.5(8.8, 14.3)	7.2(5.5, 9.1)
Selenium, μg/day	50.3(41.0, 62.5)	34.4(24.7, 42.6)
Copper, mg/day	2.4(2.0, 4.4)	1.1(0.9, 1.6)
Manganese, mg/day	4.3(3.6, 5.5)	3.0(2.1, 3.9)
AAA, mg/day	3783.9(2902.1, 4694.6)	3422.6(2514.9, 4367.4)
BCAA, mg/day	5866.2(4460.5, 7304.9)	5932.9(4541.8, 7166.2)
SAA, mg/day	1116.8(830.6, 1407.0)	1234.2(882.7, 1638.9)
TFA, g/day	36.1(23.5, 43.0)	36.4(22.6, 53.7)
SFA, g/day	17.0(10.9, 20.4)	14.9(9.0, 23.6)
MUFA, g/day	14.0(9.5, 17.8)	15.2(9.5, 22.3)
PUFA, g/day	3.2(2.3, 4.9)	4.8(3.7, 6.4)
MUFA (% of TFA) [a]	41.2(38.5, 42.3)	42.2(40.0, 44.8)
PUFA (% of TFA) [a]	10.0(9.1, 11.9)	13.7(10.7, 19.9)

Abbreviations: SFFQ: semiquantitative food frequency questionnaire; 24-HDRs: 24-hour dietary recalls; AAA: aromatic amino acids; BCAA: branched-chain amino acids; SAA: sulfur-containing amino acids; TFA: total fatty acids; SFA: saturated fatty acids; MUFA: monounsaturated fatty acids; PUFA: polyunsaturated fatty acids.

[a] The percentage of monounsaturated fatty acids or polyunsaturated fatty acids to total fatty acids.

Table S3. Median (interquartile ranges) food group intakes estimated by the SFFQ and the 24-HDRs by participant sources.

Food groups	IHPP-I			CCC	
	SFFQ1	SFFQ2	24-HDRs	SFFQ	24-HDRs
Refined grains, g/day	262.0(139.7, 327.3)	262.0(157.3, 341.4)	183.0(158.3, 221.7)	143.0(120.3, 177.4)	245.0(146.2, 350.2)
Whole grains, g/day	14.3(10.0, 57.1)	14.3(6.7, 35.7)	3.3(0.0, 28.7)	57.1(14.3, 85.7)	105.8(12.7, 171.7)
Tubers, g/day	57.1(14.3, 57.1)	57.1(14.3, 85.7)	33.3(15.8, 65.2)	57.1(14.3, 85.7)	25.0(10.4, 71.7)
Legumes, g/day	32.6(14.8, 65.0)	24.7(13.5, 44.7)	18.7(10.0, 23.3)	16.6(12.3, 23.5)	11.0(3.3, 19.1)
Vegetables, g/day	311.4(226.2, 372.6)	307.1(169.5, 456.9)	97.7(68.8, 143.7)	243.8(210.7, 365.2)	95.5(41.3, 196.2)
Salty vegetables, g/day	1.7(1.7, 5.0)	1.7(1.7, 5.0)	0.0(0.0, 2.5)	1.7(1.7, 1.7)	0.0(0.0, 0.0)
Mushrooms, g/day	33.6(12.1, 57.1)	35.7(11.1, 57.1)	6.7(0.0, 9.7)	12.1(10.0, 14.3)	0.0(0.0, 9.6)
Fruit, g/day	182.6(110.2, 241.0)	171.2(100.2, 237.7)	56.4(41.6, 127.8)	138.8(99.0, 238.3)	111.7(34.2, 277.7)
Red meat, g/day	71.4(41.8, 100.0)	57.1(48.9, 89.3)	60.0(30.7, 90.2)	14.3(10.0, 35.7)	5.0(0.4, 22.9)
Poultry, g/day	28.6(28.6, 46.4)	28.6(28.6, 42.9)	42.0(16.1, 71.1)	7.1(5.0, 7.1)	0.0(0.0, 0.0)
Animal organs, g/day	6.7(3.3, 9.4)	6.7(3.3, 10.0)	0.0(0.0, 5.8)	3.3(3.3, 5.8)	0.0(0.0, 1.3)
Processed meat, g/day	5.0(3.3, 7.1)	5.0(3.3, 17.9)	1.7(0.0, 10.0)	1.7(1.7, 5.0)	0.0(0.0, 0.0)
Seafood, g/day	24.0(18.0, 66.9)	33.8(23.1, 60.8)	16.3(0.0, 29.5)	14.7(11.3, 28.8)	0.0(0.0, 2.5)
Eggs, g/day	62.0(58.7, 67.3)	62.0(55.4, 80.1)	42.4(23.5, 65.8)	62.0(57.4, 62.0)	33.3(12.7, 74.5)
Dairy products, g/day	205.2(112.4, 393.3)	211.2(92.0, 314.0)	83.3(5.8, 197.3)	248.1(74.9, 312.4)	25.0(0.0, 94.6)
Nuts, g/day	2.5(1.7, 3.6)	3.6(0.8, 3.6)	2.3(0.0, 5.7)	3.6(2.5, 3.6)	4.3(0.0, 30.0)
Desserts, g/day	23.5(13.6, 43.6)	28.3(15.2, 44.6)	13.3(5.2, 21.7)	13.6(12.5, 18.9)	0.0(0.0, 11.0)
Beverages, ml/day	84.3(47.7, 131.7)	84.3(69.7, 143.5)	1.7(0.0, 83.3)	47.7(47.7, 74.8)	0.0(0.0, 0.0)

Abbreviations: IHPP-I: International Human Phenome Project (phase I); CCC: Central China Cohort; SFFQ: semiquantitative food frequency questionnaire; 24-HDRs: 24-hour dietary recalls.

Table S4. Median (interquartile ranges) nutrient intakes estimated by the SFFQ and the 24-HDRs by participant sources.

Nutrients	IHPP-I			CCC	
	SFFQ1	SFFQ2	24-HDRs	SFFQ	24-HDRs
Total energy, kcal/day	1979.6(1706.0, 2322.6)	1821.1(1610.7, 2281.2)	1403.2(1156.7, 1805.2)	1724.9(1366.8, 2055.7)	1971.8(1558.5, 2286.4)
Carbohydrate, g/day	220.5(183.6, 293.5)	213.1(174.1, 289.2)	163.0(125.9, 211.7)	205.2(161.0, 248.6)	252.4(183.0, 301.3)
Fat, g/day	81.1(63.2, 104.6)	76.1(59.6, 88.9)	53.7(45.8, 75.6)	80.4(56.3, 94.0)	74.3(58.8, 103.4)
Protein, g/day	88.3(71.1, 101.5)	82.4(67.6, 95.8)	69.9(58.2, 80.2)	58.6(47.7, 73.1)	60.4(42.3, 66.9)
Fiber, g/day	14.2(11.1, 18.3)	14.8(10.8, 19.9)	7.6(6.1, 10.9)	12.7(10.5, 16.0)	10.9(8.7, 13.3)
Cholesterol, mg/day	546.9(476.8, 708.1)	544.3(466.5, 712.5)	185.3(115.5, 257.7)	451.7(388.2, 492.2)	371.5(169.3, 577.2)
Vitamin A, μgRE/day	1334.5(826.3, 1497.1)	1322.7(843.5, 1508.5)	330.8(205.3, 477.6)	829.7(719.5, 1252.7)	278.5(188.1, 394.5)
Total carotene, μg/day	2493.3(1836.4, 3225.9)	2701.7(1728.9, 3376.2)	758.9(367.1, 1696.5)	2072.5(1614.7, 2830.4)	855.8(356.5, 2374.3)
Retino1, mg/day	1006.3(562.8, 1160.7)	1015.8(550.2, 1192.7)	143.6(86.2, 258.7)	575.7(476.6, 915.9)	58.0(22.1, 88.5)
Thiamin, mg/day	1.0(0.9, 1.2)	1.0(0.8, 1.1)	0.6(0.5, 0.7)	0.8(0.7, 1.0)	0.7(0.4, 1.0)
Riboflavin, mg/day	1.7(1.4, 2.3)	1.6(1.5, 1.9)	0.9(0.6, 1.1)	1.3(1.0, 1.7)	0.7(0.5, 0.8)
Niacin, mg/day	20.3(16.6, 26.8)	21.8(16.7, 26.9)	14.1(11.8, 16.4)	12.1(9.5, 14.4)	10.7(7.5, 14.2)
Vitamin C, mg/day	118.6(102.8, 159.8)	117.2(82.2, 167.5)	44.4(32.6, 75.6)	107.1(88.9, 150.9)	58.2(28.4, 99.2)
Vitamin E, mg/day	30.7(23.8, 44.0)	26.1(20.2, 32.6)	13.7(10.4, 18.6)	38.5(26.0, 49.6)	17.4(10.3, 27.1)
Calcium, mg/day	1033.1(844.8, 1276.1)	961.5(806.8, 1111.5)	438.9(342.7, 558.8)	885.5(656.4, 1065.4)	347.0(272.8, 463.6)
Phosphorus, mg/day	1421.2(1260.2, 1825.1)	1375.7(1170.2, 1662.9)	971.3(683.4, 1182.0)	1125.3(867.1, 1400.2)	788.9(616.5, 959.6)
Potassium, mg/day	3165.9(2661.1, 4025.0)	3223.3(2731.7, 3801.2)	1790.0(1310.6, 2086.7)	2401.0(1891.8, 3008.2)	1467.7(1292.0, 1809.5)
Sodium, mg/day	7381.2(6917.4, 7740.6)	7262.6(5373.2, 7724.2)	1697.7(1454.1, 2156.8)	7355.3(6992.3, 7473.6)	560.5(344.2, 881.7)
Magnesium, mg/day	406.9(344.1, 498.2)	416.3(349.1, 479.2)	206.6(167.2, 274.4)	341.0(284.2, 404.2)	276.2(183.3, 318.6)
Iron, mg/day	30.6(28.0, 35.5)	30.7(26.0, 35.3)	13.7(11.6, 16.9)	23.0(20.2, 30.6)	16.3(10.8, 19.4)
Zinc, mg/day	13.6(10.9, 16.2)	13.5(10.4, 15.1)	7.4(6.7, 9.9)	8.8(8.3, 12.0)	6.5(4.7, 8.8)
Selenium, μg/day	60.0(46.2, 67.7)	55.3(46.1, 65.9)	34.4(24.8, 40.7)	42.5(35.6, 50.9)	34.4(24.6, 43.7)
Copper, mg/day	4.5(2.2, 6.8)	2.9(2.1, 4.5)	0.9(0.8, 1.1)	2.2(1.8, 3.1)	1.6(1.1, 1.9)

Manganese, mg/day	4.8(3.7, 5.6)	5.0(3.8, 5.9)	2.9(2.1, 3.6)	4.0(3.3, 4.9)	3.3(2.1, 4.6)
AAA, mg/day	3937.1(3214.7, 5639.1)	3878.6(2966.4, 4962.8)	3258.7(2442.8, 3828.4)	3512.9(2753.7, 4403.5)	3983.8(2904.6, 4872.2)
BCAA, mg/day	7155.1(5697.4, 10340.2)	7213.1(5589.7, 9289.6)	5938.4(4615.5, 7028.9)	4798.2(3656.8, 5809.7)	5709.4(4333.9, 7129.9)
SAA, mg/day	1284.2(1167.1, 1833.4)	1403.0(1042.1, 1671.9)	1162.3(883.3, 1395.3)	872.6(692.1, 1124.5)	1300.3(889.0, 1720.2)
TFA, g/day	45.7(35.2, 58.8)	41.4(33.1, 51.1)	24.4(20.9, 32.4)	23.3(17.7, 38.7)	54.0(42.1, 80.7)
SFA, g/day	20.5(17.3, 27.6)	18.7(15.7, 24.2)	9.7(7.9, 14.2)	11.9(9.4, 17.3)	23.3(16.6, 29.5)
MUFA, g/day	18.3(13.1, 23.0)	16.6(13.7, 21.5)	10.8(8.1, 14.6)	9.2(6.3, 14.3)	22.1(19.1, 32.1)
PUFA, g/day	5.2(3.3, 6.6)	4.5(3.2, 5.5)	4.3(2.7, 5.4)	2.3(1.6, 2.9)	6.0(4.6, 11.4)
MUFA (% of TFA) [a]	40.6(38.7, 42.4)	41.7(40.4, 42.9)	41.8(39.5, 43.2)	38.5(35.5, 41.7)	42.8(40.4, 46.2)
PUFA (% of TFA) [a]	10.9(9.3, 12.9)	10.3(9.3, 12.5)	15.2(11.8, 19.7)	9.8(8.8, 10.7)	12.1(9.7, 21.8)

Abbreviations: SFFQ: semiquantitative food frequency questionnaire; 24-HDRs: 24-hour dietary recalls; AAA: aromatic amino acids; BCAA: branched-chain amino acids; SAA: sulfur-containing amino acids; TFA: total fatty acids; SFA: saturated fatty acids; MUFA: monounsaturated fatty acids; PUFA: polyunsaturated fatty acids.

[a] The percentage of monounsaturated fatty acids or polyunsaturated fatty acids to total fatty acids.

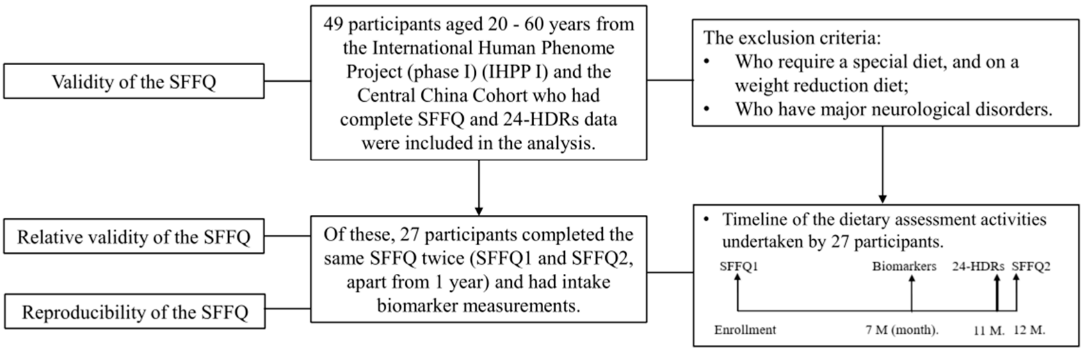

Figure S1. Design of the validation study. 49 eligible participants had complete data on semiquantitative food frequency questionnaire (SFFQ) and 24-hour dietary records (24-HDRs) for three days. A subgroup of 27 participants had plasma biomarker measurements relating to fatty acids and repeated SFFQ data (SFFQ1 and SFFQ2, apart from 1 year).

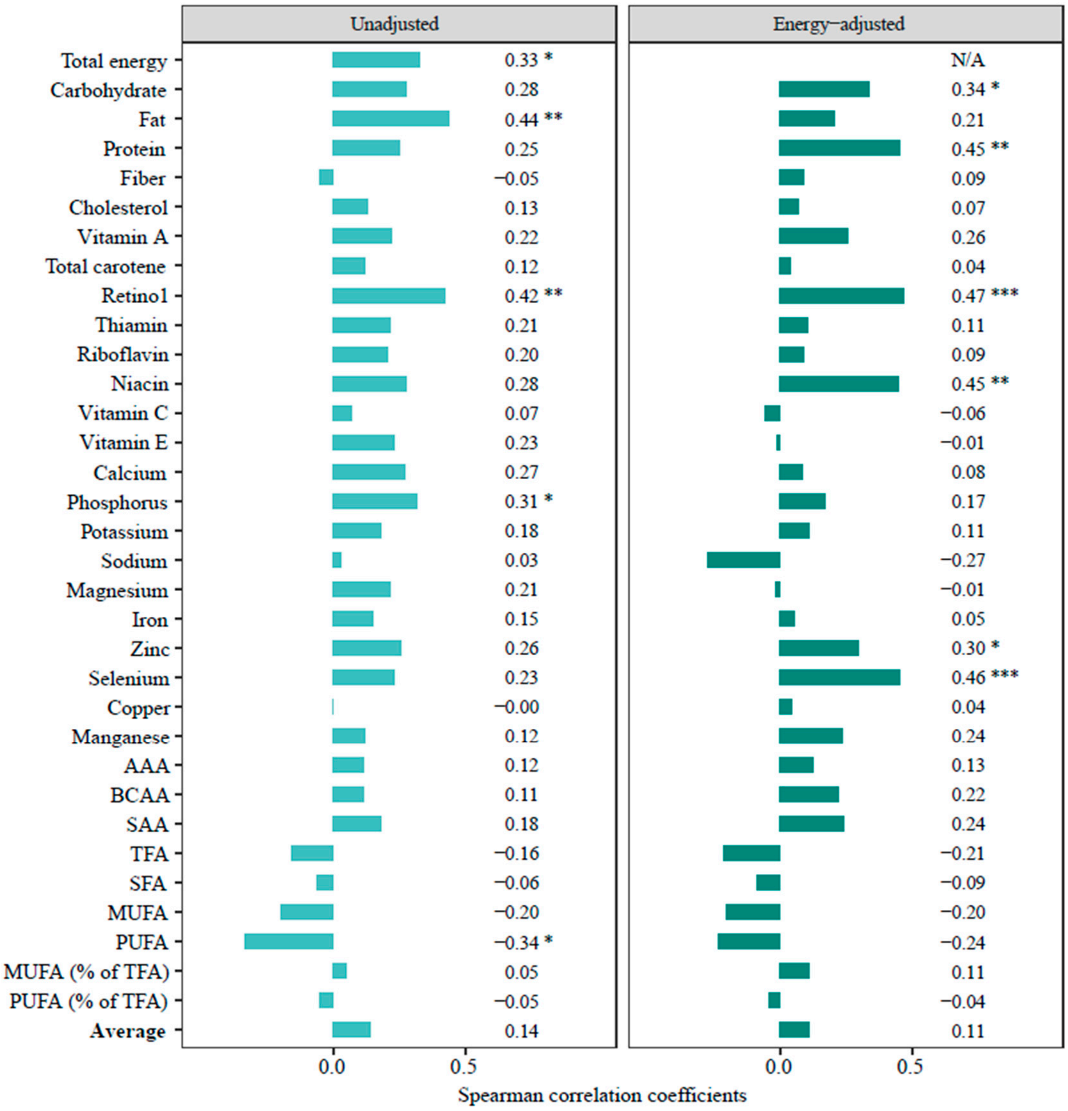

Figure S2. Spearman correlation coefficients of nutrient intakes estimated by the SFFQ and the 24-HDRs. Energy-adjusted: Total energy intake was adjusted. * $P < 0.05$; ** $P < 0.01$; *** $P < 0.001$. AAA: aromatic amino acids; BACC: branched-chain amino acids; SAA: sulfur-containing amino

acids; TFA: total fatty acids; SFA: saturated fatty acids; MUFA: monounsaturated fatty acids, PUFA: polyunsaturated fatty acids; MUFA (% of TFA): the percentage of monounsaturated fatty acids to total fatty acids; PUFA (% of TFA): the percentage of polyunsaturated fatty acids to total fatty acids.

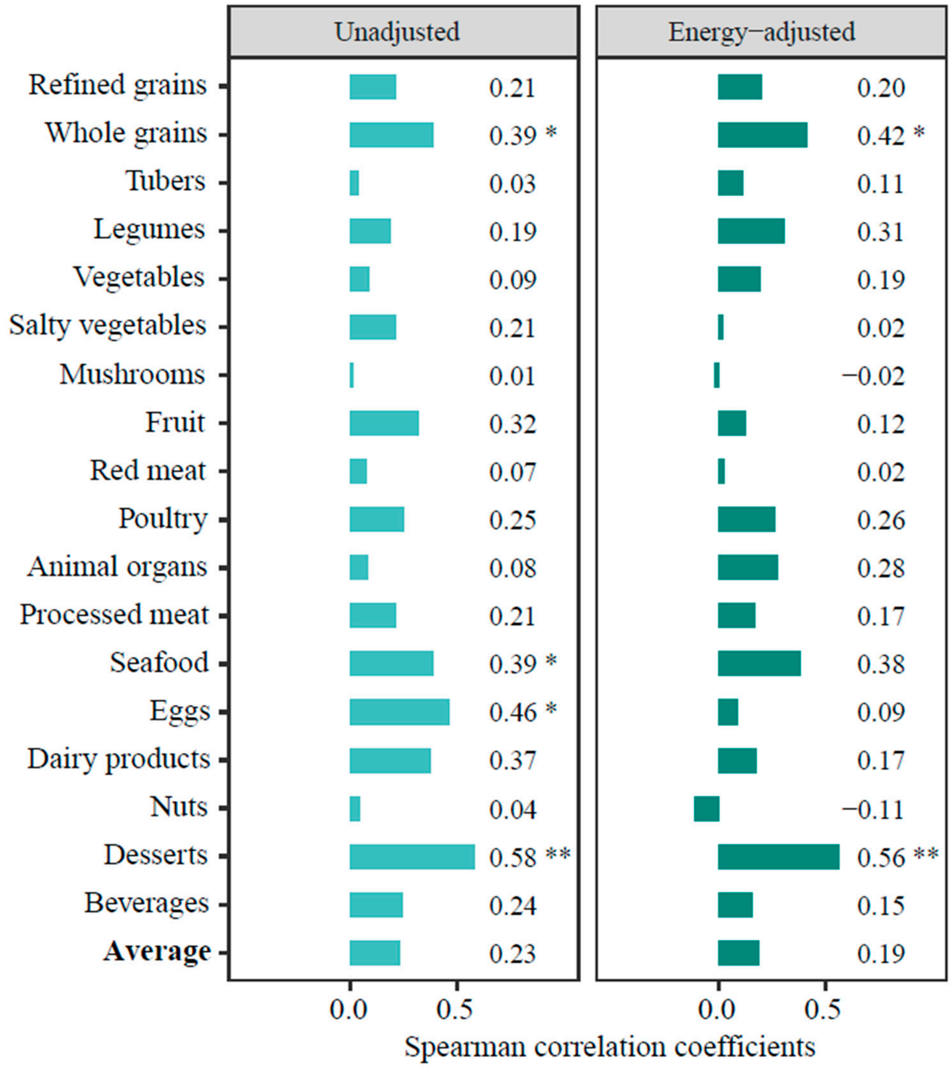

Figure S3. Spearman correlation coefficients of food group intakes estimated by the SFFQ and the 24-HDRs in the International Human Phenome Project (phase I). Energy-adjusted: Total energy intake was adjusted. * $P < 0.05$; ** $P < 0.01$.

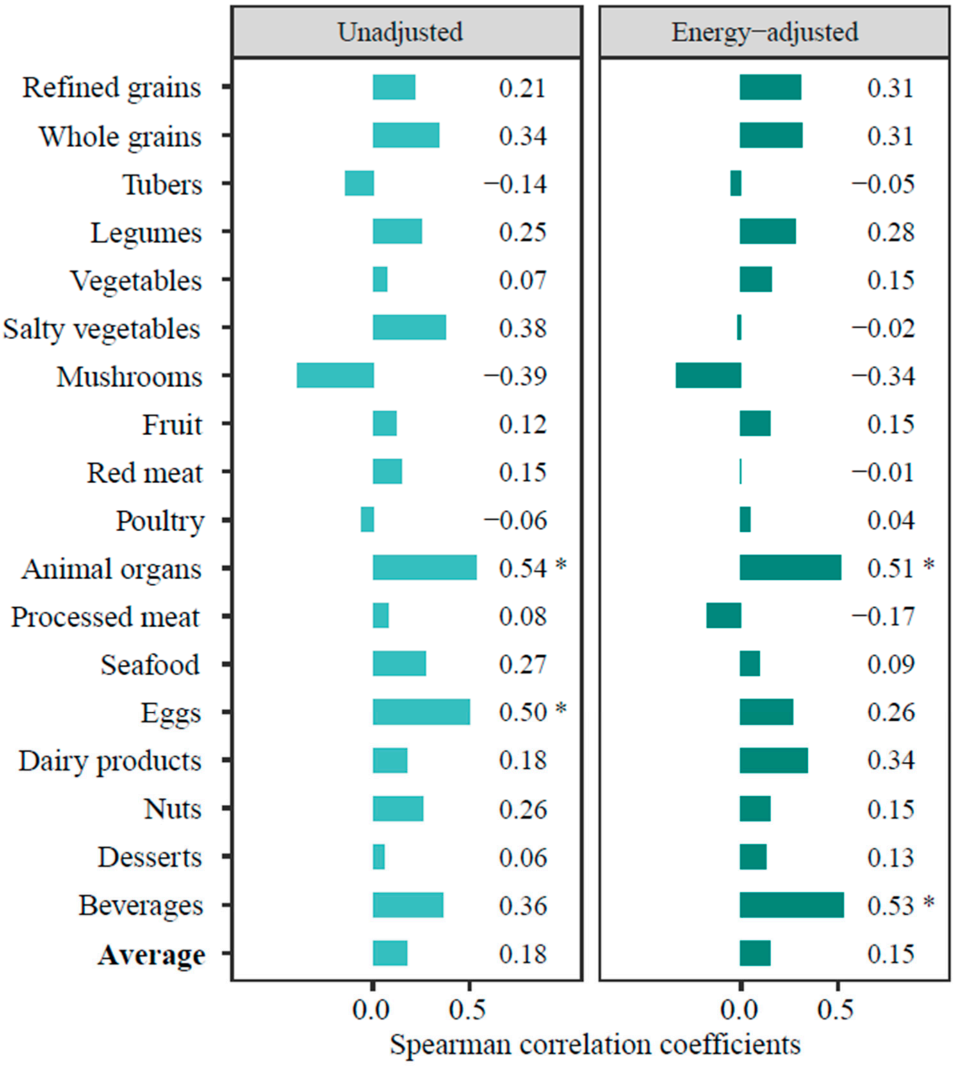

Figure S4. Spearman correlation coefficients of food group intakes estimated by the SFFQ and the 24-HDRs in the Central China Cohort. Energy-adjusted: Total energy intake was adjusted. * $P < 0.05$.

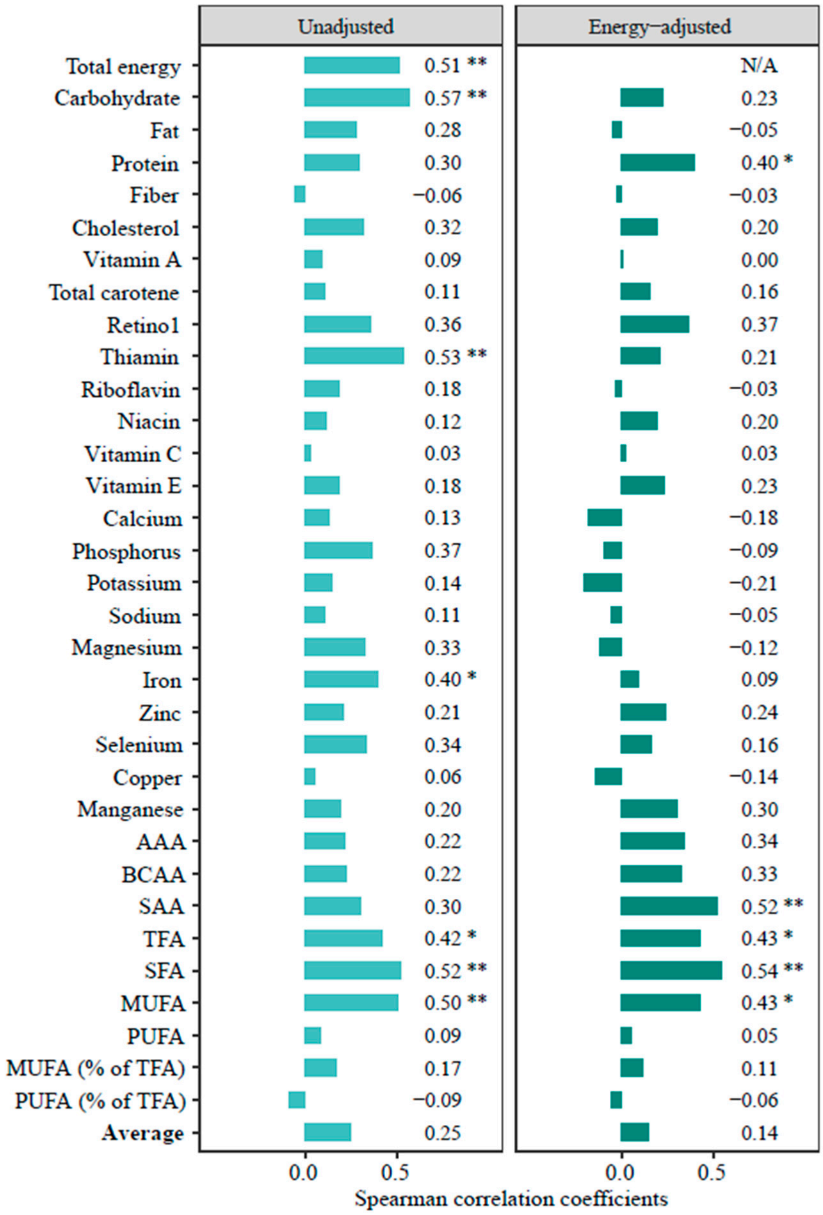

Figure S5. Spearman correlation coefficients of nutrient intakes estimated by the SFFQ and the 24-HDRs the International Human Phenome Project (phase I). Energy-adjusted: Total energy intake was adjusted. * $P < 0.05$; ** $P < 0.01$. AAA: aromatic amino acids; BACC: branched-chain amino acids; SAA: sulfur-containing amino acids; TFA: total fatty acids; SFA: saturated fatty acids; MUFA: monounsaturated fatty acids; PUFA: polyunsaturated fatty acids; MUFA (% of TFA): the percentage of monounsaturated fatty acids to total fatty acids; PUFA (% of TFA): the percentage of polyunsaturated fatty acids to total fatty acids.

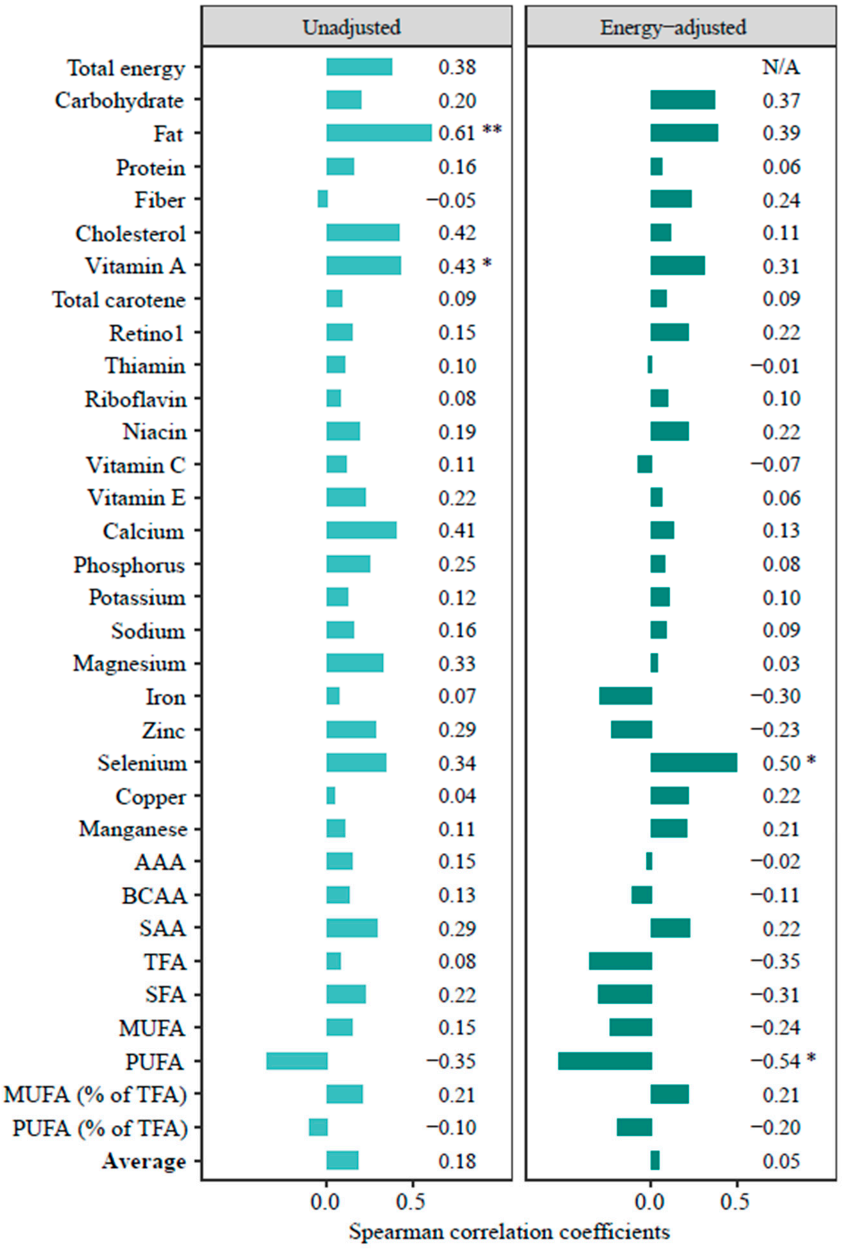

Figure S6. Spearman correlation coefficients of nutrient intakes estimated by the SFFQ and the 24-HDRs the Central China Cohort. Energy-adjusted: Total energy intake was adjusted. * $P < 0.05$; ** $P < 0.01$. AAA: aromatic amino acids; BACC: branched-chain amino acids; SAA: sulfur-containing amino acids; TFA: total fatty acids; SFA: saturated fatty acids; MUFA: monounsaturated fatty acids; PUFA: polyunsaturated fatty acids; MUFA (% of TFA): the percentage of monounsaturated fatty acids to total fatty acids; PUFA (% of TFA): the percentage of polyunsaturated fatty acids to total fatty acids.

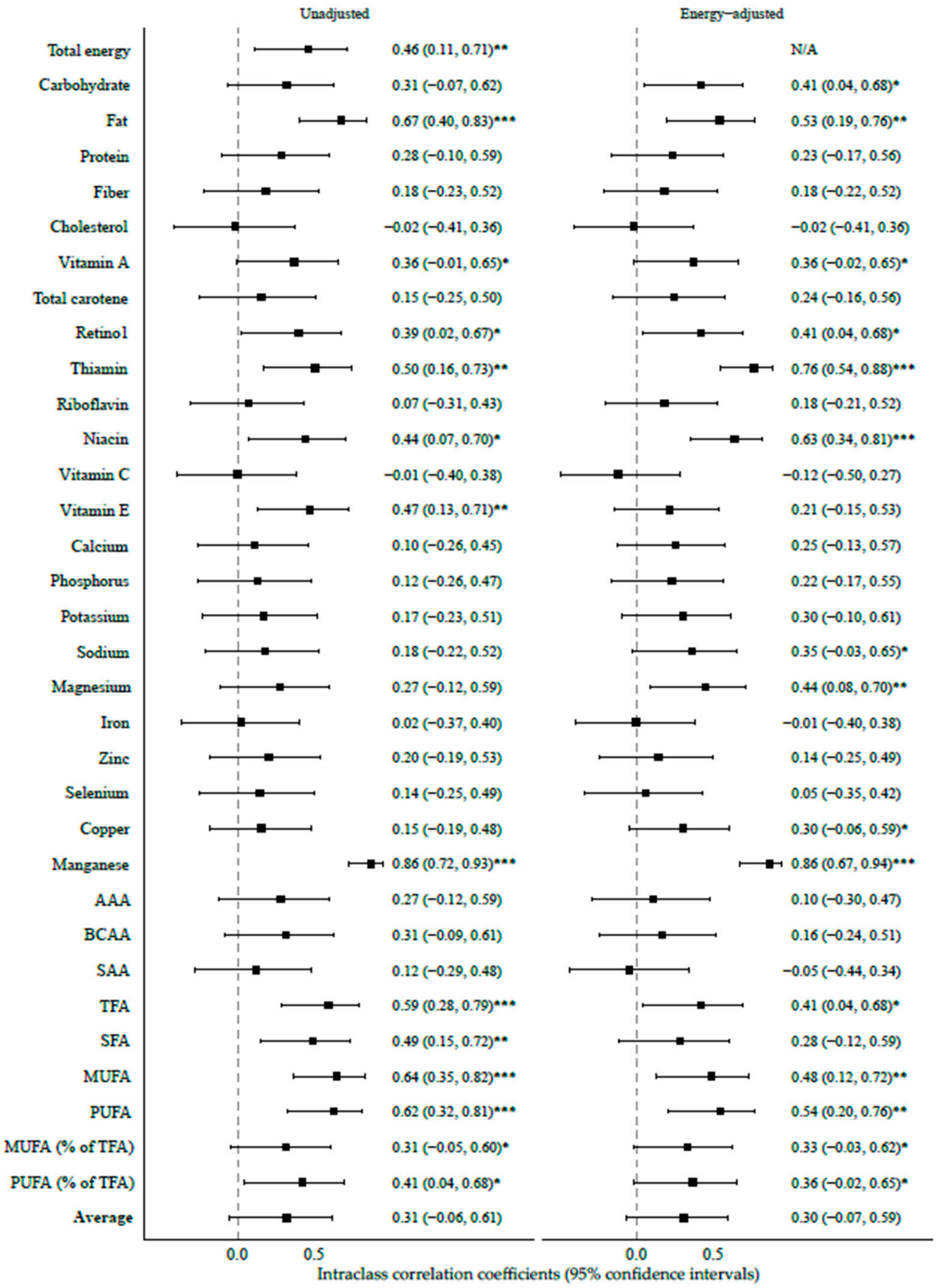

Figure S7. Intraclass correlation coefficients (ICCs) and 95% confidence intervals of nutrient intakes estimated by the SFFQ in the International Human Phenome Project (phase I). * $P < 0.05$; ** $P < 0.01$; *** $P < 0.001$. AAA: aromatic amino acids; BACC: branched-chain amino acids; SAA: sulfur-containing amino acids; TFA: total fatty acids; SFA: saturated fatty acids; MUFA: monounsaturated fatty acids; PUFA: polyunsaturated fatty acids; MUFA (% of TFA): the percentage of monounsaturated fatty acids to total fatty acids; PUFA (% of TFA): the percentage of polyunsaturated fatty acids to total fatty acids.
